# Clinical Characteristics Associated with Adherence and Persistence in Patients with Type 2 Diabetes Mellitus Treated with Dulaglutide

**DOI:** 10.1155/2023/7917641

**Published:** 2023-06-01

**Authors:** David Seung U. Lee, Howard Lee

**Affiliations:** ^1^Department of Molecular Medicine and Biopharmaceutical Sciences, Graduate School of Convergence Science and Technology, Seoul National University, Seoul 08826, Republic of Korea; ^2^Center for Convergence Approaches in Drug Development, Graduate School of Convergence Science and Technology, Seoul National University, Seoul 08826, Republic of Korea; ^3^Department of Clinical Pharmacology and Therapeutics, Seoul National University College of Medicine and Hospital, Seoul 03080, Republic of Korea; ^4^Advanced Institute of Convergence Technology, Suwon 16229, Republic of Korea

## Abstract

**Aims:**

This study is aimed at identifying clinical characteristics associated with adherence and persistence in patients with type 2 diabetes mellitus (T2DM) treated with dulaglutide.

**Materials and Methods:**

This retrospective observational cohort study used the Common Data Model at Seoul National University Hospital, Seoul, South Korea. Eligible subjects were followed for one year. Multivariate logistic and linear regressions were used to identify the factors associated with categorical (i.e., adherence status and continuation status) and continuous (i.e., proportion of days covered, or PDC, and treatment duration) outcome measures, respectively. Subgroup analysis was conducted involving patients at high cardiovascular disease (CVD) risk (i.e., having ≥2 identifiable risk factors).

**Results:**

A total of 236 patients were included. Increase in age and estimated glomerular filtration rate significantly increased the likelihood of adherence and treatment continuation. In contrast, baseline obesity and baseline use of sulfonylurea and insulin significantly reduced the likelihood of continuing dulaglutide. Similarly, increase in age, switching dulaglutide dose, and baseline neuropathy significantly increased PDC and treatment duration. None of the adherence or persistence outcome measures were significantly different between patients at high CVD risk and their matched controls. Baseline hypertension and the higher baseline LDL-C level significantly increased the likelihood of adherence in patients at high CVD risk.

**Conclusion:**

Clinical characteristics of dulaglutide users that could have affected their adherence and persistence were identified. Physicians treating T2DM patients with dulaglutide can refer to those clinical characteristics identified in this study to optimize the adherence and persistence to dulaglutide.

## 1. Introduction

Dulaglutide is a glucagon-like peptide-1 receptor agonist (GLP-1RA) indicated for the treatment of type 2 diabetes mellitus (T2DM). GLP-1RAs are administered by subcutaneous injection except for oral semaglutide. As with many injectable therapies, GLP-1RAs are prone to medication nonuse [1], which manifests in two patterns: (1) missed medication doses (nonadherence) and (2) abrupt discontinuation or substantial medication gap (nonpersistence) [2]. For example, when adherence was assessed using the average proportion of days covered (PDC) or the number of days covered by prescription fills divided by the total number of days [3], PDC for injectable GLP-1RAs at six months was only 0.61-0.76 [4], up to 20% lower than 0.8, a PDC of optimal treatment adherence. Furthermore, the proportion of nonpersistent patients with injectable GLP-1RA in six months ranged between 26.0% and 67.9% [2].

Dulaglutide has demonstrated significantly higher adherence and persistence rates than other GLP-1RAs [1, 5–7]. Additionally, a recent claims-based study has found that patients treated with dulaglutide were significantly more adherent and persistent than those treated with oral semaglutide at six-month follow-up [8]. Nevertheless, the adherence and persistence rates in dulaglutide users still fell short of being optimal (mean PDC 0.76 and 37% discontinuation rate) [4].

Optimizing treatment adherence and persistence is an important determinant of clinical outcome [9]. In this sense, it is beneficial to investigate which clinical characteristics are associated with increased adherence and persistence. However, such analysis on dulaglutide users remain understudied. Previous studies mostly limited their scopes to comparative purposes, with the goal of showing higher adherence and persistence in dulaglutide users than users of other antidiabetic medications or other GLP-1RAs [1, 4–8, 10]. Moreover, most of the previous studies have used claims data, assembled primarily for reimbursement purposes and therefore not providing important clinical data such as laboratory test results. There are also concerns about inaccuracy and incompleteness of information in claims data due to the lack of billing codes for some conditions or upcoding comorbidities [11, 12].

The objective of this study was to identify clinical characteristics associated with adherence and persistence in T2DM patients treated with dulaglutide. To this end, we used the electronic medical records (EMR) extracted, transformed, and loaded into the Common Data Model (CDM) at Seoul National University Hospital (SNUH), Seoul, South Korea.

## 2. Materials and Methods

### 2.1. Data Source

We used the Observational Medical Outcomes Partnership Common Data Model (OMOP CDM, version 5.3.1) of Seoul National University Hospital (SNUH), Seoul, South Korea. SNUH is a university-affiliated, tertiary-care hospital. The OMOP CDM of SNUH contains over 2.3 billion medical records of more than 3 million patients, including patient demographics, diagnosis, drug exposures, laboratory test orders and results, surgeries, family histories, and past medical histories [13, 14]. Since we did not collect or use individually identifiable data, the SHUH Institutional Review Board (IRB) granted a waiver for obtaining informed consent (IRB No. E-2105-137-1219).

### 2.2. Study Subjects

This was a retrospective observational cohort study. Eligible patients were those who were diagnosed with T2DM and initiated treatment with once-weekly dulaglutide (0.75 mg or 1.5 mg) between January 1, 2018, and December 31, 2019. The index date was defined as the first date of dulaglutide prescription with ≥6 months of identifiable past clinical history (i.e., baseline). Each eligible patient was followed for one year after the index date. We excluded patients if they were <18 years of age at the index date, without ≥1 record of baseline HbA1c, diagnosed with type 1 diabetes or gestational diabetes, or with a record of bariatric surgery. Additionally, patients who were lost to follow-up (i.e., without clinical history) were considered disenrolled from SNUH and therefore excluded.

### 2.3. Clinical Characteristics

We collected information on demographics, comorbidities, concomitant antidiabetic medications, and laboratory test results at baseline. We converted baseline comorbidities originally recorded in SNOMED CT into corresponding International Classification of Diseases 10^th^ Revision (ICD-10) codes by using Interactive Map-Assisted Generation of ICD Codes (I-MAGIC) [15]. After conversion, baseline comorbidities were categorized into composite events by using the diagnosis designation of the ICD-10 codes. Likewise, we grouped individual concomitant antidiabetic medications according to drug class. In addition, adverse events (AEs) were defined as any of the following conditions during follow-up: nausea, vomiting, diarrhea, indigestion, abdominal pain, lower abdominal pain, foot ulcer, impaired fasting glucose, hyperglycemia, hypoglycemia, gastroparesis, and pancreatitis [16]. Finally, we imputed values for laboratory test results and demographics with <15% missing data using the multiple imputation with chained equation (MICE) [17], whereas those with ≥15% missing data were excluded from the analysis [18].

### 2.4. Outcome Measures

Adherence was measured by PDC and adherence status. Adherence status was a categorical variable, in which patients with ≥0.8 PDC were classified as adherent and those with <0.8 PDC were nonadherent. Similarly, persistence was assessed using treatment duration and continuation status. Treatment duration represents the number of days on treatment without discontinuation (i.e., >60 days gap between any two consecutive prescriptions). Continuation status was a categorical variable, in which patients were classified as either continuer or discontinuer based on the operational definition of discontinuation. If patients had overlapping days' supply, we disregarded residual supply from the previous fill.

### 2.5. Statistical Analysis

We used multivariate linear regression and multivariate logistic regression to identify the factors associated with continuous and categorical outcome measures, respectively. Important independent variables were selected per the highest adjusted *R*^2^ value and the lowest Akaike information criterion (AIC) for the linear regression and the logistic regression models, respectively.

Because dulaglutide is also indicated for the treatment of T2DM patients with cardiovascular disease (CVD) risks, we conducted a subgroup analysis involving patients at high CVD risk. Patients with ≥2 identifiable CVD risk factors (Table S1) were defined as subjects at high CVD risk [19, 20]. The cutoff value of 2 identifiable CVD risk factors was determined based on the median number of CVD risk factors in the study subjects (Figure S1). We used propensity score (PS) to match those at high CVD risk with controls (i.e., the subjects with <2 identifiable CVD risk factor) based on baseline characteristics (i.e., demographics, comorbidities, concomitant medications, and lab test results). We allowed matching with replacement because there were not enough controls to fully provide one-to-one match. On matched cohorts, we conducted the Student's *t*-test and chi-squared (*χ*^2^) test to analyze differences between CVD riskers and nonriskers in continuous and categorical outcome measures, respectively. For treatment duration, we conducted log-rank test, in which event was defined as discontinuing dulaglutide.

To determine the robustness of the results, we conducted sensitivity analyses by (1) changing the permissible prescription gap for continuous treatment to >90 days and (2) defining the subjects at high CVD risk as having ≥3 identifiable CVD risk factors.

A *p* value < 0.05 was considered statistically significant. Statistical analysis was conducted using R (version 4.2.1).

## 3. Results

### 3.1. Subjects

**Figure 1:**
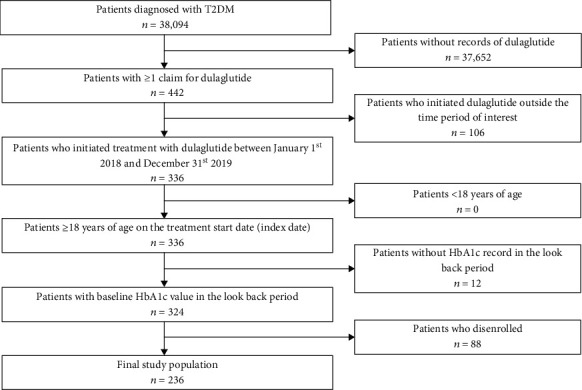
Flowchart for the study population selection. Abbreviations: T2DM: type 2 diabetes mellitus; HbA1c: glycated hemoglobin A1c.

A total of 38,094 patients with T2DM were identified, of whom 236 patients were eligible for our study (Figure 1). The mean age was 55.5 years with sex being evenly distributed (50.4% male), and dyslipidemia was the most frequent baseline comorbidity (44.9%) followed by hypertension (37.7%) (Table 1). A total of 169 (71.6%) subjects had ≥2 risk factors for CVD at baseline. More than two-thirds or 76.6% of patients (*n* = 181) initiated treatment with low-dose (0.75 mg) dulaglutide (Table 1). Furthermore, 41.1% of patients (*n* = 97) switched dose after treatment initiation with dulaglutide, among whom 92.8% (*n* = 90) switched to high dose (1.5 mg) (Table 2). Dulaglutide was well tolerated; <1% of subjects experienced one or more predefined AEs except abdominal pain (1.3%) (Table 2).

### 3.2. Adherence

**Figure 2:**
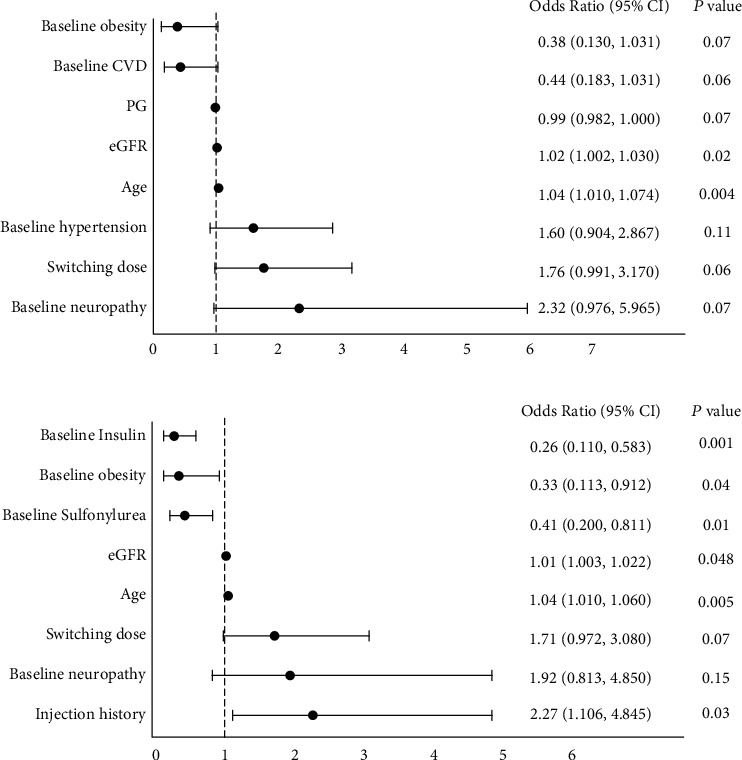
Factors affecting adherence status (a) and continuation status (b) of all subjects (*n* = 236). *p* values were determined by multivariate logistic regression. Abbreviations: 95% CI: 95% confidence interval; CVD: cardiovascular disease; PG: postprandial glucose; eGFR: estimated glucose filtration rate (CKD-EPI).

The mean PDC was 0.6, and 48.7% of subjects were adherent (Table 2). Increase in age, switching dose, and having neuropathy at baseline significantly increased PDC (*β*-coefficients [95% confidence interval or CI]: 0.006 [0.002, 0.010], 0.09 [0.003, 0.18], and 0.14 [0.01, 0.27], respectively; all *p* < 0.05) (Table 3). In contrast, baseline uses of sulfonylurea or insulin significantly decreased PDC (*β*-coefficients [95% CI]: -0.13 [-0.23, -0.022] and -0.11 [-0.21, -0.005], respectively). On the other hand, subjects were 4% more likely adherent as age increased (odds ratio or OR [95% CI]: 1.04 [1.01, 1.07], *p* < 0.05). Moreover, increase in estimated glomerular filtration rate (eGFR) was significantly associated with increased adherence (OR [95% CI]: 1.02 [1.002, 1.03], *p* < 0.05) (Figure 2).

### 3.3. Persistence

The mean treatment duration was 236.8 days, and 50.4% of subjects were continuously treated with dulaglutide during follow-up (Table 2). Increase in age, switching dose, and having neuropathy at baseline significantly increased treatment duration by 2.17 days (95% CI: 0.78, 3.55 days), 32.9 days (95% CI: 0.81, 64.9 days), and 50.6 days (95% CI: 2.94, 98.3 days), respectively (all *p* < 0.05) (Table 3). In contrast, baseline uses of sulfonylurea or insulin significantly reduced treatment duration (*β*-coefficients [95% CI]: -43.6 days [-83.2, -8.80 days] and -38.9 days [-76.1, -1.68 days], respectively; both *p* < 0.05). On the other hand, subjects who had experience with injectable therapies were over twice more likely to continue treatment than those who did not (OR [95% CI]: 2.27 (1.10, 4.84), *p* < 0.05) (Figure 2). Furthermore, subjects were significantly more likely to be continuously treated as age increased (OR [95% CI]: 1.04 [1.01, 1.06], *p* < 0.05) (Figure 2). Contrastingly, subjects using sulfonylurea or insulin or who had obesity at baseline were significantly less likely to continue treatment with dulaglutide (OR [95% CI]: 0.41 (0.20, 0.81), 0.26 (0.11, 0.58), and 0.33 (0.11, 0.91), respectively; with all *p* < 0.05). Those results in adherence and persistence did not significantly change in the sensitivity analysis (Table S2).

### 3.4. Effect of CVD Risk on the Adherence and Persistence to Dulaglutide

**Figure 3:**
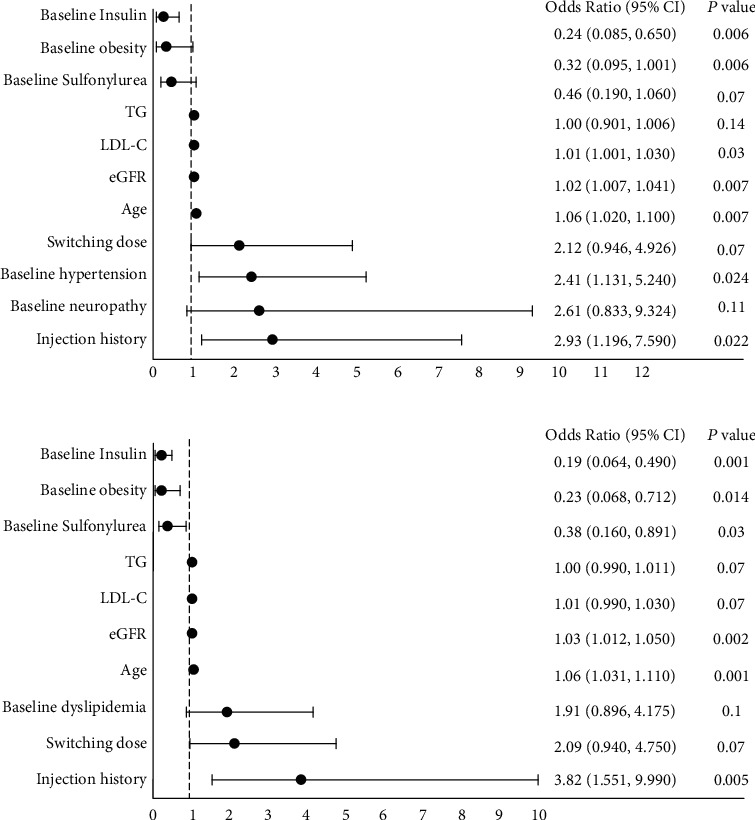
Factors affecting adherence status (a) and continuation status (b) of subjects with 2 or more CVD risk(s) (*n* = 169). *p* values determined by multivariate logistic regression. Abbreviations: 95% CI: 95% confidence interval; CVD: cardiovascular disease; TG: triglyceride; HbA1c: glycated hemoglobin A1c; LDL-C: low-density lipoprotein cholesterol; eGFR: estimated glomerular filtration rate (CKD-EPI).

**Figure 4:**
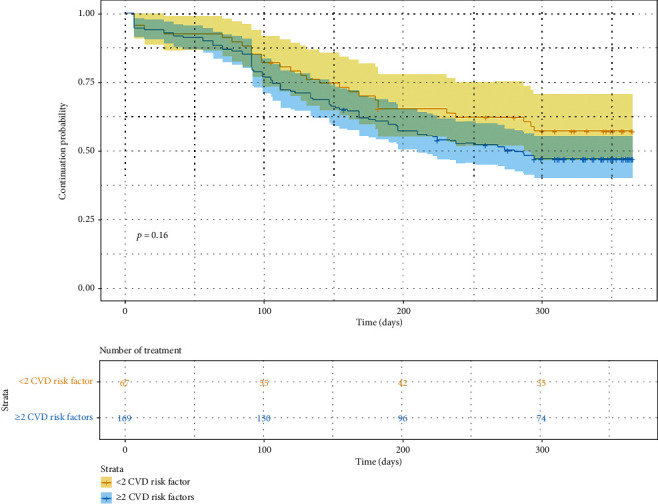
Kaplan-Meier curve for the comparison of time to treatment discontinuation on the matched cohorts between subjects with <2 CVD risk factor (*n* = 67) and subjects with ≥2 CVD risks (*n* = 169). Median was 280 days for subjects with ≥2 CVD risks and was not reached for subjects with <2 CVD risk factor. *p* value was determined by log-rank test (*χ*^2^ = 2, 1 degree of freedom). Abbreviations: CVD: cardiovascular disease.

We were able to match 67 controls with 169 subjects at high CVD risk (Table S3). None of the adherence or persistence outcome measures were significantly different between those at high CVD risk and their matched controls (all *p* > 0.05) (Table S4). The outcome measures of subjects at high CVD risk were affected by similar factors, while the presence of baseline hypertension and the higher baseline LDL-C level significantly increased the likelihood of adherence in those at high CVD risk (Figure 3). Moreover, the result of log-rank test showed that there was no significant difference in the time to discontinue dulaglutide between the matched cohorts (*p* = 0.16) (Figure 4). Sensitivity analysis comparing subjects with ≥3 CVD risks to controls showed no significant difference in the results (Table S5 and Figure S2).

## 4. Discussion

We found that the dulaglutide adherence in the study subject was not optimal. Moreover, only one half of the subjects (50.4%) continued treatment with dulaglutide for one year. These results are consistent with the findings of the previous studies [2, 4, 5, 21], which reported the adherence and persistence rates of injectable antidiabetic medications including dulaglutide as suboptimal.

Importantly, we found that several characteristics were associated with the adherence and persistence to dulaglutide treatment. Most notably, increase in age significantly improved PDC, treatment duration, and the likelihood of adherence and continuation (Figure 2 and Table 3). The results were consistent with those from the previous studies, which have identified older age as a significant predictor of adherence and persistence in T2DM patients treated with antidiabetic medications [22–25]. Older age is known to be associated with increasing severity of illness and greater awareness of health status, which can lead to higher adherence and persistence rates as seen in this and previous studies [26]. This finding is reassuring given that polypharmacy and increasing susceptibility to AEs and complications in older populations may undermine treatment adherence and persistence [27].

In addition, we found that changing the treatment dose of dulaglutide significantly improved PDC and treatment duration (Table 3). Previous studies have found that patients who initiated the low-dose (0.75 mg) dulaglutide and then switched to the high dose (1.5 mg) were significantly more likely to be adherent and persistent [6, 28]. Of note, dose switching in this study considered both escalation and deescalation of dulaglutide dose. Nevertheless, over 90% of the subjects who had switched dose underwent dose escalation. In this sense, the finding of this study was consistent with the previous findings. Gastrointestinal AEs in dulaglutide-treated patients were increased in a dose-dependent manner, which could potentially undermine adherence and persistence [29]. However, the results of this study showed that dulaglutide was well tolerated overall. Thus, dose escalation may improve rather than undermine the adherence and persistence of dulaglutide users despite the potentially higher risk of gastrointestinal AEs.

Furthermore, we found that baseline neuropathy significantly increased both PDC and treatment duration (Table 3). This finding was consistent with that from a previous study investigating insulin adherence and persistence in T2DM patients, where patients with neuropathy were more likely to be persistent [30]. As of now, more real-world evidence has to be established about the efficacy of dulaglutide on managing neuropathic comorbidities and its impact on dulaglutide adherence and persistence. Nevertheless, this finding leads to a speculation that the higher PDC and treatment duration in the subjects with neuropathy can be attributed to the once-weekly dosing interval of dulaglutide, which offers an added benefit of convenience. Neuropathic comorbidities are known to complicate routine tasks of diabetes management (e.g., checking blood glucose level) because of exaggerated pain response [31]. In this sense, the once-weekly dosing of dulaglutide may reduce the frequency of such tasks in T2DM patients with baseline neuropathy [32], contributing to improved adherence and persistence. Another explanation is that patients with baseline neuropathy are more likely to have longer T2DM duration, greater disease severity, and more failed previous treatments, which could collectively heighten their awareness of health status and thus improve their adherence and persistence.

We also found that higher baseline eGFR was associated with significantly higher likelihood of dulaglutide adherence and continuation (Figure 2). It is unlikely that this association is due to the pharmacokinetic profile of dulaglutide. Dulaglutide is composed of two GLP-1 analogues fused to a modified IgG4 Fc fragment by a small peptide link [29]. Due to the large molecular size, dulaglutide is not cleared by the kidney, and no clinically relevant difference in the pharmacokinetics (e.g., total clearance) of dulaglutide was observed in T2DM patients with impaired kidney function [29]. Instead, it may be suspected that factors external to dulaglutide, such as higher medical cost in T2DM patients with impaired kidney function [33], may have affected dulaglutide adherence and persistence. However, a further investigation is warranted. Renal protective effects of GLP-1RAs including dulaglutide, which are known to reduce protein kinase C, oxidative stress, and inflammatory response, have been well established [34, 35]. Moreover, in clinical studies, treatment with dulaglutide was associated with a significantly smaller decline in eGFR or reduced composite renal outcomes than comparators and placebo [36, 37]. An analysis of integrated data from 9 phase II and III trials of dulaglutide has also found that treatment with dulaglutide decreased albuminuria and was not associated with an increase in AEs reflecting potential acute renal failure [38]. Considering T2DM is the leading cause of chronic kidney disease [39] and eGFR typically declines by approximately 2 to 4 mL/min a year in T2DM patients [38, 40], the renal protective effect of dulaglutide can greatly benefit the patients with low kidney function. Therefore, attention must be paid to such patients to improve treatment adherence and persistence and eventually treatment outcome.

We found that the presence of obesity at baseline significantly reduced the likelihood of dulaglutide continuation (Figure 2). The weight benefit of GLP-1 RAs including dulaglutide has been established in randomized clinical trials (RCTs) [41, 42]. For example, patients treated with 1.5 mg dulaglutide over 26 weeks achieved a clinically meaningful weight loss (mean bodyweight change from baseline: -2.9 kg) [43]. However, previous real-world studies have reported a significant heterogeneity in the magnitude of weight loss in GLP-1 RA users, a substantial proportion of whom underwent no significant change in bodyweight [44, 45]. Treatment effect observed in RCTs often exceeds the real-world effectiveness due in part to insufficient representativeness of clinical trial participants [46] or greater accessibility to resources and support systems that help comply with treatment regimen during RCTs [28]. Considering that clinical improvement may improve treatment adherence and persistence [47], the efficacy-effectiveness gap in the weight benefit of dulaglutide may have led to the significantly lower likelihood of continuing dulaglutide in subjects with baseline obesity, despite the purported weight benefit of dulaglutide.

In the subgroup analysis, we found that the dulaglutide adherence and persistence were not significantly different for a large portion of subjects who had a high CVD risk (Table S4 and Figure 4). This result may be attributed to the large portion of the study subjects having high CVD risk (*n* = 169 or 71.6%). Furthermore, it may be speculated that the comparable adherence and persistence rates in dulaglutide users at high CVD risk may be due to the potential delay of the CVD preventive effect of dulaglutide. Cardiovascular benefits of dulaglutide and their durability, particularly in middle-aged or older T2DM patients, are well established [48]. However, underlying metabolic abnormalities that eventually lead to CVDs may remain asymptomatic for years before clinical manifestation [49, 50]. Similarly, the CVD preventive effect of a medication may become apparent over an extended period of time. Such delay may have prevented the CVD benefits of dulaglutide from translating into improved dulaglutide adherence and persistence, at least within a year. On the other hand, the set of clinical factors associated with the adherence and persistence of dulaglutide users with high CVD risk was comparable to those of all subjects (Figure 3 and Table 3). Of note, we found that in subjects with high CVD risk, the presence of baseline hypertension and the higher baseline LDL-C level significantly increased the likelihood of adherence. However, a further investigation is warranted to ascertain whether such phenomenon is due to the experience of clinical improvement or an expectation for improvement.

Previous studies have found that one of the reasons for discontinuing dulaglutide is AEs like gastrointestinal symptoms [6, 51]. The results of this study showed that AEs known to be associated with dulaglutide [52] were relatively rare in the study subjects; i.e., <1% of the study subjects experienced an AE except abdominal pain (1.3%). These results may suggest that dulaglutide was generally well tolerated, and the experience of AEs at least within a year may not significantly interfere with medication-taking behaviors in dulaglutide users. Of note, the incidence rates of AEs as reported by RCTs of dulaglutide were higher. For example, a meta-analysis of dulaglutide RCTs found that 7.8%, 11.2%, 7.3%, and 5% of the participants treated with dulaglutide experienced hypoglycemia, nausea, vomiting, and diarrhea, respectively [52]. Thus, our results for AEs should be taken with caution because transient or nonemergent symptoms could be underreported.

This study had several limitations. First, the factors found to decrease or increase the adherence or persistence to dulaglutide treatment are not necessarily causal. Of note, there were unmeasured confounders that could have affected the medication behavior in dulaglutide users. It is well established that there are multiple dimensions of factors for treatment adherence and persistence: health care-related factors (e.g., access to health care), condition-related factors (e.g., the alleviation of symptom), and therapy-related factors (e.g., ease of taking medication) but also social factors (e.g., social support and economic factors) and patient-related factors (e.g., demographics and health beliefs) [47]. Specifically, the data source of this study did not contain data on social factors and health care-related factors. Moreover, condition-related factors, patient-related factors, or therapy-related factors that are not routinely captured by EMR may not have been included in the analysis. Despite the unmeasured potential confounders, we used electronic medical records of a tertiary university hospital and provided a higher granularity information on the factors for dulaglutide adherence and persistence, including CVD risks. Secondly, this was a single-center study with a relatively small number of patients. This study used EMR from a tertiary university hospital, in which patients with greater disease severity are more likely to be treated, leading to a potential selection bias. However, the results of this study on dulaglutide adherence and persistence were similar to those of the previous studies which used national claims data. Finally, this study was conducted on the assumption that the decision to adhere to and continue the treatment with dulaglutide is largely patient-oriented. In the analysis, it was not possible to ascertain the extent to which the decision to continue (or discontinue) dulaglutide was driven by physicians or patients. However, by employing four distinct outcome measures, the impact of such uncertainty may have been mitigated. Since persistence (as measured by treatment duration and continuation status) can be more prone to such uncertainty, adherence (as measured by PDC and adherence status), which describes the density or sparseness of prescription filling records while on treatment, may be more appropriate for evaluating the medication-taking behavior in T2DM patients treated with dulaglutide.

## 5. Conclusion

In conclusion, clinical characteristics of dulaglutide users that could have affected their adherence and persistence were identified, which were generally comparable to the reports of the previous studies. Specifically, increase in age and estimated glomerular filtration rate significantly increased the likelihood of adherence and treatment continuation. In contrast, baseline obesity and baseline use of sulfonylurea and insulin significantly reduced the likelihood of continuing dulaglutide. Similarly, increase in age, switching dulaglutide dose, and baseline neuropathy significantly increased PDC and treatment duration. While none of the adherence or persistence outcome measures were significantly different between patients at high CVD risk and their matched controls, baseline hypertension and the higher baseline LDL-C level significantly increased the likelihood of adherence in patients with a high CVD risk. Physicians treating T2DM patients with dulaglutide can refer to those clinical characteristics identified in this study to fine-tune their approaches to optimize the adherence and persistence to dulaglutide and possibly to other antidiabetic medications, not only before but during the treatment.

## Figures and Tables

**Table 1:** Baseline characteristics of the total study population and subjects with ≥2 CVD risk(s).

Variables	Total (*n* = 236)	Subjects with ≥2 identifiable CVD risk factors (*n* = 169)
Sex		
Male, *n* (%)	119 (50.4%)	76 (45.0%)
Female, *n* (%)	117 (49.6%)	93 (55.0%)
Age at index date, mean (SD)	55.5 (13.7)	55.2 (14.1)
Baseline lab test results		
HbA1c, % (SD)	8.3 (1.4)	8.2 (1.5)
Systolic BP, mmHg (SD)	132.1(15.8)	134.4(16.4)
Diastolic BP, mmHg (SD)	80.0 (11.3)	81.7 (11.6)
Total cholesterol, mg/dL (SD)	158.7 (37.2)	157.1 (37.5)
LDL, mg/dL (SD)	86.5 (30.4)	89.6 (31.1)
HDL, mg/dL (SD)	47.1 (12.1)	45.0 (11.1)
Triglyceride, mg/dL (SD)	171.8 (105.3)	187.6 (114.5)
eGFR (MDRP), mL/min/1.73 m^2^ (SD)	85.6 (27.0)	86.8 (26.5)
eGFR (CKDEPI), mL/min/1.73 m^2^ (SD)	88.5 (25.1)	85.9 (26.5)
Postprandial glucose, mg/dL (SD)	158.6 (54.2)	159.1 (54.6)
Starting dose		
0.75 mg, *n* (%)	181 (76.7%)	129 (76.3%)
1.5 mg, *n* (%)	55 (23.3%)	40 (23.7%)
Baseline concomitant antidiabetic medication		
Metformin, *n* (%)	217 (91.9%)	157 (94.1%)
Insulin, *n* (%)	88 (37.3%)	66 (39.1%)
Meglitinide, *n* (%)	1 (0.4%)	0 (0%)
DPP4 inhibitor, *n* (%)	42 (17.8%)	29 (17.2%)
SGLT2 inhibitor, *n* (%)	46 (19.5%)	33 (19.5%)
Alpha glucosidase, *n* (%)	2 (0.8%)	2 (1.2%)
Thiazolidinedione, *n* (%)	9 (3.8%)	4 (2.4%)
Sulfonylurea, *n* (%)	149 (63.1%)	101 (59.8%)
DPP4 inhibitor plus metformin combination drug, *n* (%)	50 (21.2%)	38 (22.5%)
SGLT2 inhibitor plus metformin combination drug, *n* (%)	3 (1.3%)	2 (1.2%)
Sulfonylurea plus metformin combination drug, *n* (%)	6 (2.5%)	4 (2.4%)
Pioglitazone plus DPP4 inhibitor combination drug, *n* (%)	2 (0.8%)	1 (0.6%)
Injection history	134 (56.8%)	102 (60.4%)
Previously treated with insulin, *n* (%)	126 (53.4%)	96 (56.8%)
Previously treated with GLP-1RA other than dulaglutide, *n* (%)	28 (11.9%)	24 (14.2%)
Baseline comorbidity		
Hypertension, *n* (%)	89 (37.7%)	84 (49.7%)
Obesity, *n* (%)	21 (8.9%)	21 (12.4%)
Dyslipidemia, *n* (%)	106 (44.9%)	83 (49.1%)
Cardiovascular disease, *n* (%)	29 (12.3%)	26 (15.4%)
Kidney disease, *n* (%)	50 (21.2%)	38 (22.5%)
Eye disease, *n* (%)	72 (30.5%)	49 (29.0%)
Neuropathy, *n* (%)	29 (12.3%)	20 (11.8%)
Mental or memory impairment, *n* (%)	10 (4.2%)	10 (5.9%)
Disease history		
Previously diagnosed with myocardial infarction, *n* (%)	12 (5.1%)	9 (5.3%)
Previously diagnosed with heart failure, *n* (%)	4 (1.7%)	3 (1.8%)
Previously diagnosed with lesion in thyroid, *n* (%)	23 (9.7%)	13 (7.7%)
CVD risk at baseline		
Low (<2 CVD risk factor(s))	67 (28.4%)	0 (100%)
High (≥2 CVD risk factors)	169 (71.6%)	169 (0%)

SD: standard deviation; PDC: proportion of days covered; HbA1c: glycated hemoglobin A1c; BP: blood pressure; LDL: low-density lipoprotein; HDL: high-density lipoprotein; eGFR: estimated glomerular filtration rate; MDRP: modification of diet in renal disease; CVD: cardiovascular disease CKDEPI: chronic kidney disease epidemiology collaboration; DPP4: dipeptidyl peptidase 4; SGLT2: sodium glucose cotransporter 2; GLP1-RA: glucagon-like peptide 1 receptor agonist.

**Table 2:** Treatment adherence and persistence results.

Variables	Total (*n* = 236)	Subjects with ≥2 identifiable CVD risk factor(s) (*n* = 169)
Continuation status		
Continued, *n* (%)	119 (50.4%)	80 (47.3%)
Discontinued, *n* (%)	117 (49.6%)	89 (52.7%)
Treatment duration, mean days (SD)	236.8 (124.9)	230.5 (125.0)
PDC, mean (SD)	0.6 (0.3)	0.63 (0.34)
Adherence, *n* (%)		
Yes (PDC ≥ 0.8)	115 (48.7%)	78 (46.2%)
No (PDC < 0.8)	121 (51.3%)	91 (53.8%)
Switching		
Yes, *n* (%)	97 (41.1%)	63 (37.3%)
1.5 mg to 0.75 mg in 1^st^ switching	7 (7.2%)	4 (6.3%)
0.75 mg to 1.5 mg in 1^st^ switching	90 (92.8%)	59 (93.7%)
No, *n* (%)	139 (58.9%)	106 (62.7%)
Adverse events		
Nausea, *n* (%)	1 (0.4%)	0 (0%)
Vomiting	0 (0.0%)	0 (0.0%)
Diarrhea	0 (0.0%)	0 (0.0%)
Indigestion, *n* (%)	2 (0.8%)	1 (0.6%)
Abdominal pain, n (%)	3 (1.3%)	3 (1.8%)
Lower abdominal pain, *n* (%)	1 (0.4%)	1 (0.6%)
Hyperglycemia, *n* (%)	2 (0.8%)	2 (1.2%)
Hypoglycemia, *n* (%)	1 (0.4%)	0 (0%)
Impaired fasting glucose, *n* (%)	1 (0.4%)	1 (0.6%)
Foot ulcer, *n* (%)	1 (0.4%)	0 (0%)
Gastroparesis, *n* (%)	1 (0.4%)	1 (0.6%)
Pancreatitis, *n* (%)	0 (0.0%)	0 (0.0%)

SD: standard deviation; PDC: proportion of days covered.

**Table 3:** Factors affecting PDC and treatment duration.

	PDC			Treatment duration		
	Factor	*β*-Coefficient (95% CI)	*p* value	Factor	*β*-Coefficient (95% CI)	*p* value
All patients (*n* = 236)	Age	0.006 (0.002, 0.010)	0.002	Age	2.17 (0.78, 3.55)	0.002
	eGFR	0.002 (-0.0003, 0.0003)	0.11	eGFR	0.55 (-0.12, 1.23)	0.11
	Switching dose	0.09 (0.003, 0.18)	0.04	Switching dose	32.9 (0.81, 64.9)	0.04
	Baseline insulin	-0.11 (-0.21, -0.005)	0.04	Baseline insulin	-38.9 (-76.1, -1.68)	0.04
	Baseline sulfonylurea	-0.13 (-0.23, -0.022)	0.02	Baseline sulfonylurea	-43.6 (-83.2, -8.08)	0.02
	Baseline obesity	-0.12 (-0.27, 0.03)	0.12	Baseline obesity	-43.4 (-98.3, 11.5)	0.12
	Baseline neuropathy	0.14 (0.01, 0.27)	0.04	Baseline neuropathy	50.6 (2.94, 98.3)	0.04
	Baseline CVD	-0.11 (-0.24, 0.03)	0.12	Baseline CVD	-39.2 (-88.4, 10.0)	0.12

Patients with ≥2 identifiable CVD risk(s) (*n* = 169)	Age	0.006 (0.001, 0.011)	0.009	Age	2.23 (0.54, 3.91)	0.001
	Male sex	0.079 (-0.023, 0.180)	0.129	Male sex	28.48 (-8.69, 65.66)	0.132
	TG	0.0004 (-0.00008, 0.0009)	0.103	TG	0.147 (-0.03, 0.323)	0.103
	eGFR	0.0028 (0.0006, 0.0049)	0.015	eGFR	1.017 (0.205, 1.830)	0.014
	Switching dose	0.1304 (0.0022, 0.243)	0.019	Switching dose	48.2 (7.74, 88.66)	0.020
	Baseline insulin	-0.143 (-0.277, -0.0089)	0.037	Baseline insulin	-52.24 (-101.28, -3.20)	0.037
	Baseline sulfonylurea	-0.158 (-0.275, -0.0416)	0.008	Baseline sulfonylurea	-57.75 (-100.31, -15.19)	0.008
	Injection history	0.0905 (-0.037, 0.219)	0.166	Injection history	32.92 (-13.99, 79.83)	0.168
	Baseline neuropathy	0.1415 (-0.0145, 0.297)	0.075	Baseline neuropathy	51.57 (-5.44, 108.58)	0.076
	Baseline obesity	-0.1453 (-0.300, 0.0094)	0.065	Baseline obesity	-53.02 (-109.54, 3.52)	0.066

PDC: proportion of days covered; eGFR: estimated glomerular filtration rate; CVD: cardiovascular disease; TG: triglyceride; *p* value determined by the multivariate linear regression after variables were removed from the model using backward selection method.

## Data Availability

The data that support the findings of this study are not publicly available due to privacy or ethical restrictions.

## Supplementary Materials

Supplementary Materials Table S1: list of identifiable risk factors for CVD. Table S2: sensitivity analysis results (using cutoff as 90-day prescription gap). Table S3: comparison of baseline characteristics between matched cohorts. Table S4: comparative analysis of endpoints in patients with and without high CVD risk. Table S5: comparative analysis of endpoints in patients with and without high CVD risk (sensitivity analysis). Figure S1: distribution of number of identifiable risk factors for cardiovascular disease (CVD). Figure S2: the Kaplan-Meier curve for the comparison of time to treatment discontinuation on the matched cohorts between subjects with <3 CVD risk (*n* = 159) and subjects with ≥3 CVD risk(s) (*n* = 77). Median was not reached for subjects with ≥3 CVD risks and was 292 days for subjects with <3 CVD risk factors. *p* value was determined by log-rank test (*χ*^2^ = 0.1, at 1 degree of freedom). Abbreviations: CVD: cardiovascular disease.
