# Supplementary material for: Clinical Characteristics Associated with Adherence and Persistence in Patients with Type 2 Diabetes Mellitus Treated with Dulaglutide
Source: J Diabetes Res. 2023 Jun 1;2023:7917641. doi: 10.1155/2023/7917641 (PMC10250096; doi:10.1155/2023/7917641)
Supplement: Supplementary Materials — Table S1: list of identifiable risk factors for CVD. Table S2: sensitivity analysis results (using cutoff as 90-day prescription gap). Table S3: comparison of baseline characteristics between matched cohorts. Table S4: comparative analysis of endpoints in patients with and without high CVD risk. Table S5: comparative analysis of endpoints in patients with and without high CVD risk (sensitivity analysis). Figure S1: distribution of number of identifiable risk factors for cardiovascular disease (CVD). Figure S2: the Kaplan-Meier curve for the comparison of time to treatment discontinuation on the matched cohorts between subjects with <3 CVD risk (n = 159) and subjects with ≥3 CVD risk(s) (n = 77). Median was not reached for subjects with ≥3 CVD risks and was 292 days for subjects with <3 CVD risk factors. p value was determined by log-rank test (χ2 = 0.1, at 1 degree of freedom). Abbreviations: CVD: cardiovascular disease. [file 7917641.f1.pdf]

SUPPLEMENTARY MATERIALS

Table S1 List of identifiable risk factors for CVD

Identifiable risk factor	Criteria
LDL-C	≥100 mg/dL
TG	≥150 mg/dL
HDL-C	<40 mg/dL (men), <50 mg/dL (women)
Blood Pressure	≥140 mmHg (systolic) and/or ≥90 mmHg (diastolic)
Baseline hypertension[†]	Diagnosis record exists at baseline
Baseline obesity	Diagnosis record exists at baseline
Baseline dyslipidemia[‡]	Diagnosis record exists at baseline
Baseline CVD	Diagnosis record exists at baseline

[†]Counted only the subjects who did not meet the criteria for blood pressure; [‡]Counted only the subjects who did not meet the criteria for LDL-C, TG, and HDL-C; Abbreviations: LDL-C, low density lipoprotein Cholesterol; TG, triglyceride; HDL-C, high density lipoprotein cholesterol; CVD, cardiovascular disease

Table S 2 Sensitivity analysis results (using cutoff as 90-day prescription gap)

Endpoint	60-day prescription gap (n=236)	90-day prescription gap (n=236)	P value
PDC, mean (SD)	0.65 (0.3)	0.67 (0.3)	0.46
Treatment duration, mean (SD)	236.8 (124.9)	244.9 (116.7)	0.46
Adherence, n (%)	115 (48.7)	115 (48.7)	0.93
Continuation, n (%)	119 (50.4)	131 (55.5)	0.27

SD, standard deviation; PDC, proportion of days covered; P value determined by Student’s t-tests for PDC and treatment duration and by χ^2 tests for adherence and continuation

Table S 3 Comparison of baseline characteristics between matched cohorts

Variable	Subjects with ≥2 identifiable CVD risk factors (n=169)	Subjects with <2 identifiable CVD risk factor (n=67)	p value
**Demographics**			
Age (years), mean (SD)	55.2 (14.1)	56.3 (12.8)	0.594
Sex (male, %)	45.0	52.2	0.120
**Laboratory test results**			
HbA1c (%), mean (SD)	8.2(1.4)	8.4 (1.4)	0.310
eGFR (mL/min/1.72 m2), mean (SD)	85.9 (26.5)	85.0 (28.4)	0.810
PG (mg/dL), mean (SD)	159.1 (54.6)	157.3 (53.7)	0.820
**Concomitant medication**			
Metformin users (%)	94.0	89.6	0.557
Insulin users (%)	39.5	32.8	0.459
DPP4 inhibitor users (%)	17.4	19.4	0.828
SGLT2 inhibitor users (%)	19.8	19.4	1.000
Sulfonylurea users (%)	60.5	71.6	0.120
**Comorbidities**			
Kidney disease (%)	22.5	17.9	0.549
Eye disease (%)	29.0	34.3	0.518
Neuropathy (%)	11.8	13.4	0.907

CVD, Cardiovascular disease; SD, standard deviation; HbA1c, glycated hemoglobin A1c; GFR, glomerular filtration rate; PG, postprandial glucose; DPP4, dipeptidyl peptidase 4; SGLT2, sodium glucose co-transporter 2; p values were calculated using Student's t-tests and χ^2 tests for continuous and categorical variables, respectively.

Table S 4 Comparative analysis of endpoints in patients with and without high CVD risk

Endpoint	Subjects with ≥2 identifiable CVD risk factors (n=169)	Subjects with <2 identifiable CVD risk factors (n=67)	P value
PDC, mean (SD)	0.63 (0.34)	0.69 (0.34)	0.219
Treatment duration, mean (SD)	230.5 (125.0)	252.6 (123.9)	0.221
Adherence, n (%)	78 (46.2)	37 (55.2)	0.210
Continuation, n (%)	80 (47.3)	39 (58.2)	0.132

SD, standard deviation; PDC, proportion of days covered; CVD, cardiovascular disease; P value determined by Student's t-tests for PDC and treatment duration and by χ^2 tests for adherence and continuation

Table S 5 Comparative analysis of endpoints in patients with and without high CVD risk (sensitivity analysis)

Endpoint	Patients with ≥3 CVD risk (n = 77)	Patients with <3 CVD risk (n = 159)	P value
PDC, mean (SD)	0.66 (0.35)	0.64 (0.34)	0.62
Treatment duration, mean (SD)	242.5 (125.6)	233.9 (124.8)	0.62
Adherence, n (%)	39 (50.6)	76 (47.8)	0.68
Continuation, n (%)	40 (51.9)	79 (49.7)	0.74

SD, standard deviation; PDC, proportion of days covered; CVD, cardiovascular disease; P value determined by Student's t-tests for PDC and treatment duration and by χ^2 tests for adherence and continuation

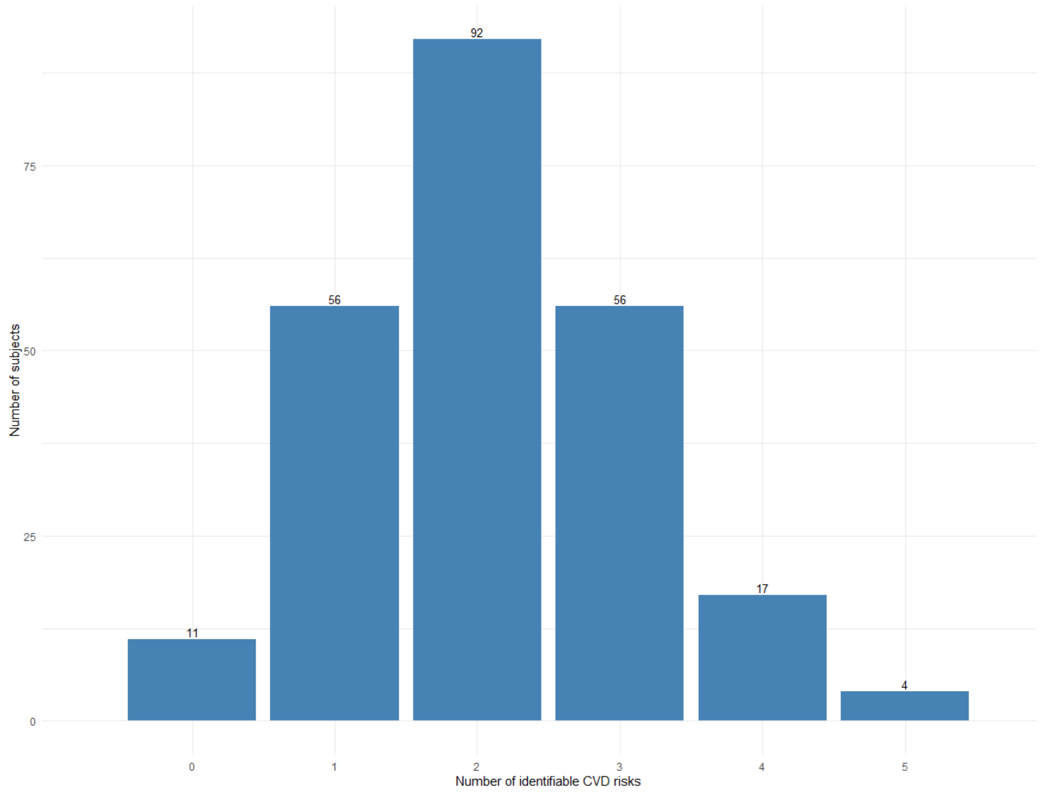

Figure S 1 Distribution of number of identifiable risk factors for cardiovascular disease (CVD)

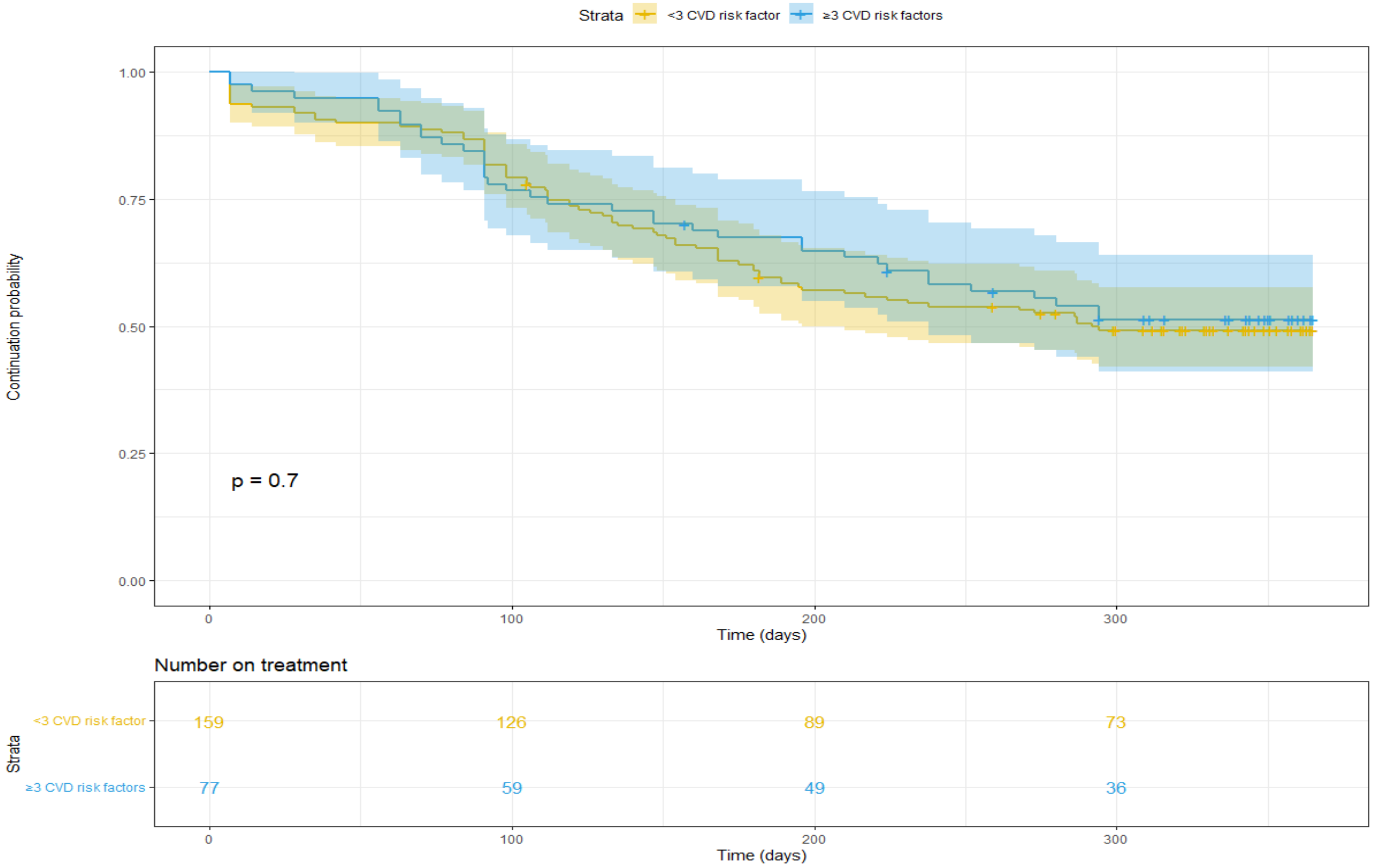

Figure S 2 Kaplan-Meier curve for the comparison of time to treatment discontinuation on the matched cohorts between subjects with 3< CVD risk (n=159) and subjects with ≥3 CVD risk(s) (n=77). Median was not reached for subjects with ≥3 CVD risks and was 292 days for subjects with <3 CVD risk factors. P value was determined by log-rank test (χ^2=0.1, at 1 degree of freedom). Abbreviations: CVD, cardiovascular disease
